# Aliphatic Polycarbonate-Based Binders for High-Loading Cathodes by Solvent-Free Method Used in High Performance LiFePO_4_|Li Batteries

**DOI:** 10.3390/ma17133153

**Published:** 2024-06-27

**Authors:** Bin Chen, Zhe Zhang, Change Wu, Sheng Huang, Min Xiao, Shuanjin Wang, Hui Guo, Dongmei Han, Yuezhong Meng

**Affiliations:** 1 School of Chemical Engineering and Technology, Sun Yat-sen University, Guangzhou 510275, China; chenb223@mail2.sysu.edu.cn (B.C.); zhangzh569@mail2.sysu.edu.cn (Z.Z.); guoh37@mail.sysu.edu.cn (H.G.); 2 The Key Laboratory of Low-Carbon Chemistry & Energy Conservation of Guangdong Province/State Key Laboratory of Optoelectronic Materials and Technologies, School of Materials Science and Engineering, Sun Yat-sen University, Guangzhou 510275, China; wuche@mail2.sysu.edu.cn (C.W.); huangsh47@mail.sysu.edu.cn (S.H.); stsxm@mail.sysu.edu.cn (M.X.); wangshj@mail.sysu.edu.cn (S.W.); 3 Institute of Chemistry, Henan Academy of Sciences, Zhengzhou 450000, China; 4 College of Chemistry, Zhengzhou University, Zhengzhou 450001, China

**Keywords:** aliphatic polycarbonate, polymer binder, lithium-ion battery, dry electrode

## Abstract

The binder ratio in a commercial lithium-ion battery is very low, but it is one of the key materials affecting the battery’s performance. In this paper, polycarbonate-based polymers with liner or chain extension structures are proposed as binders. Then, dry LiFePO_4_ (LFP) electrodes with these binders are prepared using the solvent-free method. Polycarbonate-based polymers have a high tensile strength and a satisfactory bonding strength, and the rich polar carbonate groups provide highly ionic conductivity as binders. The batteries with poly (propylene carbonate)-plus (PPC-P) as binders were shown to have a long cycle life (350 cycles under 1 C, 89% of capacity retention). The preparation of dry electrodes using polycarbonate-based polymers can avoid the use of solvents and shorten the process of preparing electrodes. It can also greatly reduce the manufacturing cost of batteries and effectively use industrial waste gas dioxide oxidation. Most importantly, a battery material with this kind of polycarbonate polymer as a binder is easily recycled by simply heating after the battery is discarded. This paper provides a new idea for the industrialization and development of a novel binder.

## 1. Introduction

In order to meet the growing demand for energy storage equipment, lithium-ion batteries, as the most popular energy storage device at present, have been widely studied. At present, the research on lithium-ion batteries mainly focuses on electrodes [1,2], electrolytes [3,4] and diaphragms [5,6]. There are relatively few studies on binders. Although binders have the least involvement in the electrodes, they play an important role in maintaining the structural integrity of the electrodes. Polyvinylidene difluoride (PVDF) is the most commonly used commercial binder. It has a good adhesion ability but it has three main problems. Firstly, in the preparation of an electrode, organic solvents, such as N-methyl-2-pyrrolidone (NMP), are needed which will pollute the environment. And the electrode drying step also occupies most of the production time and energy consumption [7,8,9]. Secondly, PVDF has insufficient ion conductivity, which will have adverse effects on the internal resistance of the electrodes. Particularly when the ion conductivity of active materials is also very low (such as LFP, the most common commercial electrode active material), the use of a binder with good ion conduction ability is much more important [10,11]. Thirdly, when the battery electrode is recovered, it needs a high temperature to decompose. Moreover, toxic gases such as HF will be produced during thermal decomposition because of the electrode containing fluorine atoms.

To solve the problems mentioned above, a polymer binder with a certain amount of Li^+^ conductivity is considered to prepare an electrode using the solvent-free method. The solvent-free method solves the problem of solvent pollution and can greatly reduce the energy loss during production [12,13,14]. For commercial electrodes, the binder content is usually no more than 5%, and the PVDF content is not more than 2%. And the reported dry electrode often has a higher load than the traditional wet electrode. So, the binder for a dry electrode requires higher adhesion (structures containing high proportion of heteroatoms) and dispersion abilities. 

**Figure 1:**
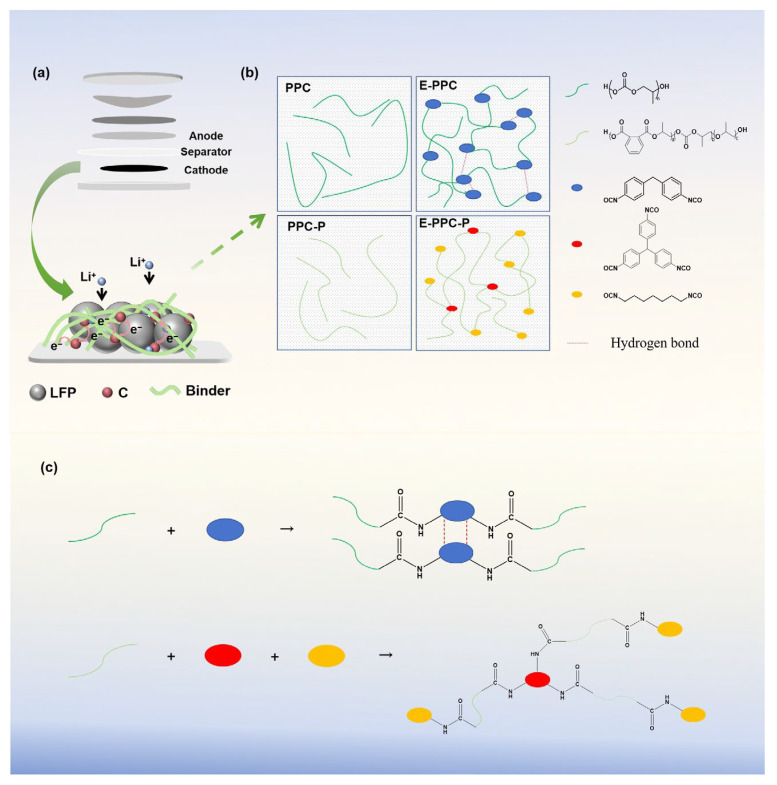
(**a**) Schematic diagram of the LFP electrode composition. (**b**) Schematic structure of the PPC-based binders. (**c**) Synthesis scheme of E-PPC and E-PPC-P.

Here, on the basis of our previous research findings [15], we used the LFP as the target electrode and used several polycarbonate-based (PPC-based) polymers as binders in combination with the solvent-free method to prepare dry LFP electrodes for LFP|Li batteries. PPC-based polymers were synthesized using industrial waste gas carbon dioxide (CO_2_) as raw material. As mentioned above, the dry electrode has higher requirements for adhesion and fluidity at high temperatures (so that the binder can be evenly dispersed between the active materials) to maintain the integrity of the high load electrode structure [16,17]. The PPC-based polymers have excellent mechanical properties (they are mostly used in the preparation of degradable plastics, films and so on [18,19], but few researches have used them as a battery binder) and excellent bonding effects (as their structures contain high proportion of heteroatoms). At the same time, the PPC-based polymers are already in the flow dynamic phase at 150 °C, and can be more evenly distributed between the electrode materials during the thermal pressure step. So, they are suitable for dry electrodes. Considering the poor Li^+^ conductivity of some active materials (such as LFP and Silicon) and binders (especially PVDF), the use of binders with a good Li^+^ conductivity capacity takes on an important role [20,21,22,23,24]. The PPC-based polymers have a good Li^+^ conductivity capacity because of the rich carbonate groups facilitating Li^+^ migration. Four polycarbonate-based polymers with different structures are tested as binders, named poly (propylene carbonate)/chain extension-poly (propylene carbonate)/poly (propylene carbonate)-plus/chain extension-poly (propylene carbonate)-plus (PPC/E-PPC/PPC-P/E-PPC-P). Figure 1a,b illustrates an LFP electrode structure using PPC-based binders. Batteries using PPC-based binders exhibit a better electrochemical performance than PVDF cells. Finally, the PPC-based binders also show advantage in the recycling of electrodes, which can be completely decomposed into CO_2_ and water when heated to 400 °C.

## 2. Materials and Methods

### 2.1. Materials

LiFePO_4_ (LFP) and conductive carbon black Super P (SP) were purchased from Canrd Co., Ltd. (Dongguan, China). Carbon nanotube (CNT, 10~20 nm) came from Solarbio Science & Technology Co., Ltd. (Beijing, China). Polyvinylidene difluoride (PVDF) was obtained from LIGE Science Co., Ltd. (Guangzhou, China); PPC was purchased from Tianguan Co., Ltd. (Nanyang, China) (Mw ≈ 25,000); PPC-P was purchased from Tianyuan Co., Ltd. (Maoming, China) (Mw ≈ 50,000). Triphenylmethane-4,4′,4′′-triisocyanate (TTI) and Hexamethylene Diisocyanate (HDI) and Methylene diphenyl diisocyanate (MDI) were purchased from Macklin Biochemical Technology Co., Ltd. (Shanghai, China).

### 2.2. Preparation of Chain Extension Binder

The chain extension of PPC and PPC-P followed the same procedure. Taking the typical preparation process of E-PPC as an example, the PPC was put into the torque rheometer (XSS-300, KeChuang Rubber Plastic Machinery Set Ltd., Shanghai, China) and preheated for 10 min at 150 °C. And then 3 wt.% MDI was added as a chain extension agent. After mixing and shearing until the torque did not change at 170 °C, a PPC with a chain extension structure was obtained and named E-PPC. The preparation of E-PPC-P is similar to that of E-PPC. In contrast, the chain extension agents used for E-PPC-P were HDI (fraction 0.5% of quality) and TTI (fraction 0.1% of quality) with a blending temperature of 150 °C. Figure 1c shows the chain expansion reaction of PPC and PPC-P. The E-PPC/E-PPC-P uses isocyanate as hard segment and PPC/PPC-P as soft segment. Due to the covalent hydrogen bonding interaction between MDI segments, E-PPC has a dynamic cross-linking structure. Because of the presence of TTI, E-PPC-P has a 3D web-like structure.

### 2.3. Preparation of Cathode Electrodes

The active material (LFP), conductive agent (mass ratio, Super P (SP)–carbon nanotube (CNT) = 2:8) and binder were premixed for 10 min at mass ratio of 88:10:2, and then we conducted ball milling at 150 rpm for 12 h to obtain a composite powder with uniform color. Later, the electrode powder was hot pressed on aluminum foil at 170 °C and 8 MPa, and then cut into circular pieces with diameter of 12 mm. The mass loading of LFP electrodes prepared by this method is about 20 mg/cm^2^.

### 2.4. Characterizations

**Figure 2:**
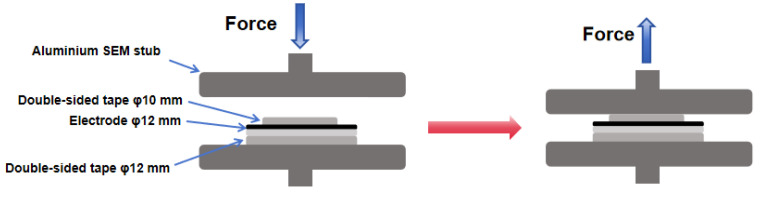
Schematic diagram of the electrode pull-off test.

The melting flow rate was tested using a MFI452 melting flow rate tester (Wance testing machine Co., Ltd., Shenzhen, China). The electrode surface morphology before and after cycle were tested using scanning electron microscope (SEM, SU8010, Hitachi, Tokyo, Japan) with an acceleration voltage of 5 kV. TGA (Perkin-Elmer, Waltham, MA, USA) was used to test the mixing uniformity of the materials with a temperature range of 40 to 650 °C and a heating rate of 10 °C/min. The DSC was tested using differential scanning calorimeter (DSC200PC Phox, Netzsch, Selb, Germany) with a temperature range of −50 to 200 °C and heating rate of 10 °C/min. A universal test machine (QJ-211, QingJi Instrumentation Technology Co., Ltd., Shanghai, China) was used to characterize the mechanical properties of the polymer binder with a test speed of 10 mm/min. The nanomechanical test instrument (Agilent Nano Indenter G200, Palo Alto, CA, USA) was used to demonstrate the internal tightness of the electrodes, with a maximum downpressure force of 10 mN. The intelligent electronic tension test machine (CG10, Labthink LanGuang, Jinan, China) was used to demonstrate the pull-off adhesion force of the binders. The pull-off adhesion method involves compression and tension phases, with the entire sequence performed on a uniaxial tensile testing machine so that precise and repeatable forces are applied [25]. We applied 3M double-sided tape to the current collector side of the coating. We adhered 3M double-sided tapes with diameters of 12 mm and 10 mm to the back and front of the electrode, respectively, and the other side of the double-sided tape was adhered to the upper and lower aluminum stub, respectively. Figure 2. shows a schematic of the complete stub, electrode and tape assembly. The electrodes were first pressed down and held for 60 s at 80 N, and then tested with a stripping speed of 100 mm/min.

To assess the electrochemical properties of the LFP electrode, coin cells (2032 coin) with PP diaphragm (Celgard 2500), Li anode and electrolyte (1.0 M LiTFSI in V% DOL: DME = 1:1 with 2% LiNO_3_) (All the materials were purchased from Canrd Co., Ltd. Dongguan, China) were assembled in the glove box (Mikrouna, Shanghai, China) under a high purity Ar atmosphere. A constant current charge/discharge test was performed for different current densities of the cathode at 28 °C and from 2.4 to 3.8 V. The charge/discharge curve was measured by the CT2001A Battery Tester System (Wuhan, China). Electrochemical impedance spectroscopy was performed using electrochemical workstation (CHI604E, Chenhua, Shanghai, China) at frequencies of 0.1 MHz to 0.1 Hz and a voltage amplitude of 5 mV. The electrochemical stability window was also determined by linear sweep voltammogram on the same electrochemical workstation. The scanning voltage range was 0–6 V with a scan speed of 1 mV/s. The effect of the binder on the cathode was evaluated using Galvanostatic intermittent titration technique (GITT) at 0.1 C. The battery underwent a repeated discharge pulse for 2 h rest until the potential decreased to 2.4 V. The following formula was used, where τ is the relaxation time, $n_{m}$ is the mole number, $V_{m}$ is the molar volume of the electrode material, S is the electrode/electrolyte contact area, $∆E_{s}$ is the voltage change caused by the pulse, and $∆E_{t}$ is the voltage change from the constant current charge/discharge.

(1) $$D=\frac{4}{\pi \tau }{\left(\frac{n_{m}V_{m}}{S}\right)}^{2}{\left(\frac{∆E_{s}}{∆E_{t}}\right)}^{2}$$

The direct current internal resistance (DC-IR) of different cathode batteries was determined. The battery was first charged to 3.5 V at 0.05 C before being left to rest for 1 h. Then, it was charged and discharged (the charge and discharge process is crossed over) at the same C rate (1/40, 1/20, 1/10, 1/5, 1/3, 1/2 C), and at the end of each process it rested for 20 min.

## 3. Results and Discussion

### 3.1. Analysis of the Physical/Chemical Properties of the Binders

**Figure 3:**
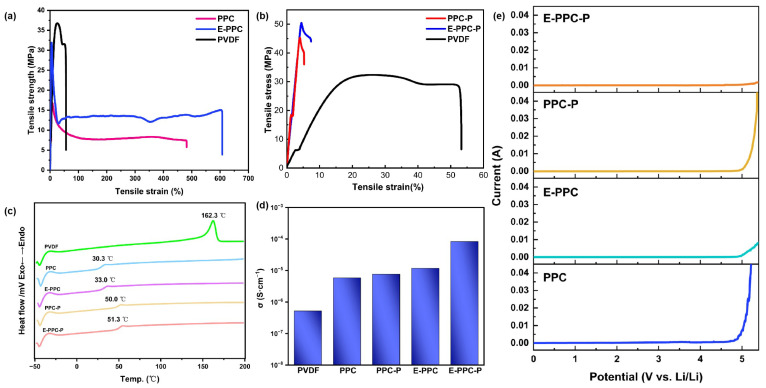
Tensile strength–strain of (**a**) PPC/E-PPC/PVDF and (**b**) PPC-P/E-PPC-P/PVDF; (**c**) DSC curves of PPC-based polymers and PVDF; (**d**) ionic conductivity of binders at 30 °C; (**e**) linear sweep voltammogram of PPC-based polymers.

The melting flow rate (MFR) of the binders was tested. At 160 °C, the MFR of PPC is 7.5 g/10 min. But at this temperature, E-PPC is poorly mobile and cannot form noodle-like samples. At 190 °C, the MFR of PPC-P is 26.0 g/10 min. However, at this temperature, E-PPC-P has poor mobility cannot form a noodle-like sample. The change in MFR is due to the increase in the average relative molecular weight between the cross-linking sites. The mechanical properties of the binders were evaluated (Figure 3a,b). At 25 °C, the tensile strength of PPC is 16.8 MPa and that of E-PPC is 31.9 MPa. The tensile strength of both is lower than PVDF (36.8 MPa), but the fracture elongation of PPC is over 480%, and the fracture elongation of E-PPC is over 605%, which are much higher than that of PVDF (56.3%). However, the tensile strengths of PPC-P (45.2 MPa) and E-PPC-P (50.4 MPa) are significantly higher than that of PVDF, while their break elongation (PPC-P: 5.3%, E-PPC-P: 7.4%) is lower than that of PVDF. In addition, the chain extension binders’ (E-PPC and E-PPC-P) tensile strength and fracture elongation have been improved compared with the PPC/PPC-P. This may be attributed to the fact that the chain extension structure will increase the intermolecular force, making the intermolecular force more stable, thus improving the strength of the material. And the extended chain connection point will inhibit the development of plastic strain, making the material under the tensile strain experience microscopic fractures. Thus, the local stress distribution is more uniform, increasing the fracture elongation to a certain extent. The improvement in the mechanical properties of the materials also confirms the existence of a chain extension structure.

The DSC curves (Figure 3c) of different polymers show that the PPC-based polymers are amorphous materials with low glass transition temperatures (PPC: 30 °C, E-PPC: 33 °C, PPC-P: 50 °C, E-PPC-P: 51 °C) and the melting point of PVDF is 162 °C. During the operation of the batteries, the PPC-based polymer binders will exist in a glassy state, which makes the cathode material be in close contact with the carbon material and the current collector. So, it can effectively resist microscopic deformation to improve the performance of the battery.

The ionic conductivity of the binders was evaluated (Figure 3d). To simulate the actual situation of the binders in the electrode, we tested the ion conductivity of the binders at 30 °C after fully infiltrating the polymer films with the electrolyte [21]. The result shows that E-PPC-P has the highest ionic conductivity (8.3 × 10^−5^ S/cm), and the ionic conductivity of the remaining PPC-based polymer binders are also significantly higher than that of PVDF (5.2 × 10^−7^ S/cm). This can be attributed to the PPC-based polymers containing abundant carbonate groups, and PPC-P/E-PPC-P containing rich benzene ring groups, which can promote the transport of Li^+^. Excellent ion conduction properties can also help to reduce the internal resistance of the electrode and improve the performance of the battery. In addition, the ionic conductivity of E-PPC/E-PPC-P is significantly improved compared with that of PPC/PPC-P.

Figure 3e shows the LSV test on the PPC-based polymer binders. The result shows that the PPC-based binders are resistant to high voltages and can maintain structural stability during 2.4–3.8 V (PPC: 4.8 V, E-PPC: 4.9 V, PPC-P: 4.9 V, E-PPC-P: 5.0 V). The result also indicates that the PPC-based polymers have the potential to act as binders for high-voltage electrodes such as NCM (generally requiring a high voltage up to 4.5 V).

### 3.2. Electrode Powder Test

**Figure 4:**
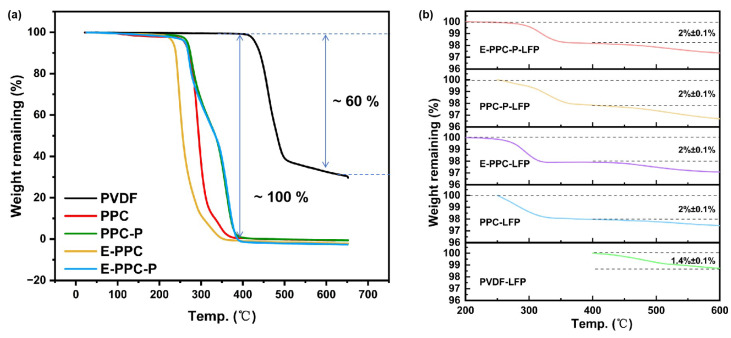
TGA of (**a**) different binders and (**b**) different electrode powders after ball milling.

The uniformity of the binder dispersion in the powder after ball milling was evaluated by a thermogravimetric analyzer in a nitrogen atmosphere [15]. As shown in Figure 4a, the PPC-based polymers start to decompose at 200–300 °C and can fully decompose at 400 °C, while the PVDF still has ~40% residue at the temperature of 600 °C. This shows that the advantages of PPC-based polymer binders can be easily removed, realizing the separation from other electrode materials, and facilitating the recycling of electrode materials when the battery is discarded. A thermogravimetric analysis was performed by random sampling multiple tests. Figure 4b shows that the binder content in the electrode material powder is consistent with the design (2%), indicating that the dispersion of the binder in the powder is uniform.

### 3.3. Adhesion Force Test

**Figure 5:**
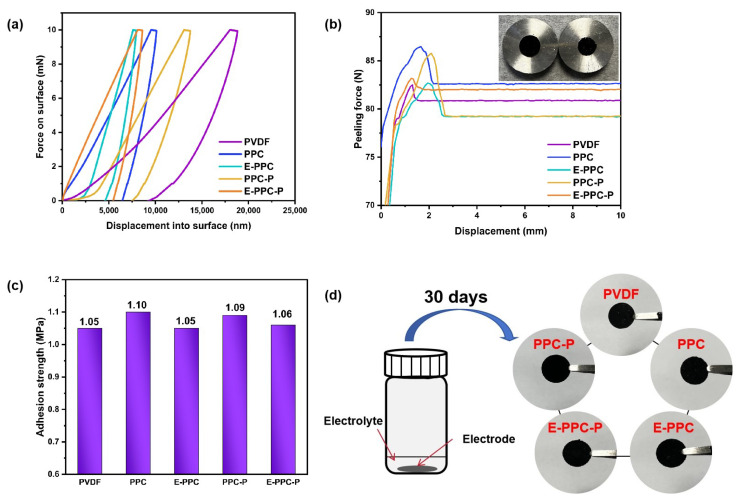
(**a**) Load indentation–depth curves; (**b**) force displacement curves during the peeling process of electrodes; (**c**) max peeling force of different electrodes. (**d**) Schematic diagram of different electrodes immersed in electrolyte for 30 days.

Figure 5a shows the typical load indentation–depth curves in the nano-indentation test. It reflects the strength of the binder adhesion force. It proves that the adhesion ability of PPC-based polymers is better than PVDF in the dry electrode. Among all the binders, E-PPC has the optimal adhesion force, followed by E-PPC-P, PPC and PPC-P. The adhesion force of polymers with chain extension structures is significantly stronger than that of their initial structures without chain extension. However, the adhesion force of PPC-P is lower than that of PPC because the presence of the rigid benzene ring in the PPC-P/E-PPC-P structure increases the steric resistance, thus reducing the intermolecular force and the content of heteroatoms such as oxygen, leading to a decrease in the adhesion force of the polymer. Figure 5b,c show the pull-off test results of the different electrodes. The test schematic diagram is shown in Figure 2. The test result also shows that the PPC-based polymers have a better adhesion ability than PVDF. However, the difference is that the adhesion ability of PPC/PPC-P electrodes is higher than that of E-PPC/E-PPC-P. This is because the mobility of binders with a chain extension structure decreases at the same temperature, which leads to a decrease in the uniformity of the binder distribution in the electrode during thermal pressing. Figure 5d shows the electrodes’ immersion in the electrolyte experiment. The electrodes were completely immersed in the electrolyte for 30 days, the electrolyte was replaced every 10 days and the surfaces of the electrode were observed and recorded. It proves that the electrode material and the current collector are closely connected in the prepared electrodes. 

### 3.4. Electrochemical Performance

**Figure 6:**
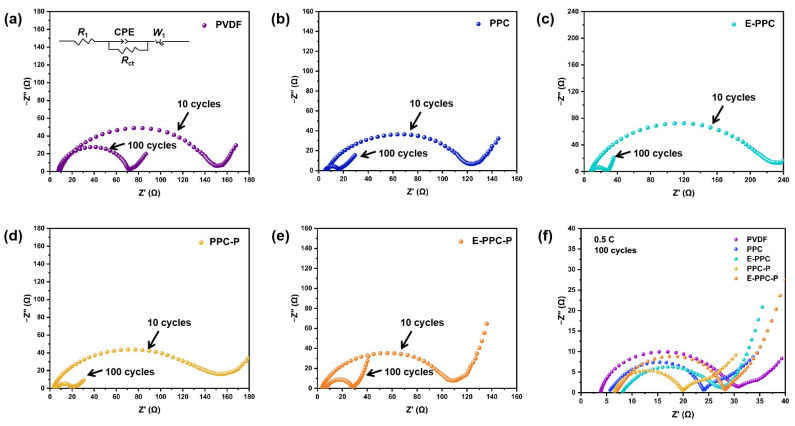
(**a**–**e**) Impedance changes in different electrodes after 10 and 100 cycles at 0.5 C. (**f**) EIS spectra of different electrodes after 100 cycles at 0.5 C.

**Figure 7:**
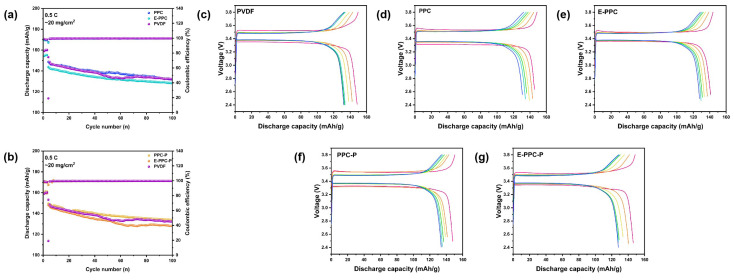
Electrochemical performances of different LFP electrodes. Cycle performance of (**a**) PPC/E-PPC/PVDF and (**b**) PPC-P/E-PPC-P/PVDF at 0.5 C; (**c**–**g**) The 5th/20th/40th/60th/80th/100th galvanostatic charge/discharge curves of different electrodes at 0.5 C.

Figure 6a–e show the impedance spectra of different electrodes and the equivalent circuit model of the Nyquist plots at 0.5 C after 10 and 100 cycles. The EIS curves of the batteries consist of a high-frequency semicircle (charge transfer resistance-R_ct_) and a low-frequency line (diffusion resistance). We found that the impedance changes in all the dry electrodes are obvious because the dry electrode structure is tight and the electrolyte infiltration is slow. After 100 cycles, the cathodes prepared with PPC-P had the lowest interface resistance, and the rest of the PPC-based electrodes all had a lower interface resistance than PVDF (Figure 6f). The PPC-based polymer binders have good ionic conductivity, and the carbonate functional groups and ester functional groups in the PPC-based polymer chain segment have good compatibility with the carbonate and ester solvent of the electrolyte, which can further improve the interface performance of the electrode. The impedance of the batteries prepared with chain-extension-structure binders are obviously greater than that prepared by polymer binders without chain extension. This is because the swelling phenomenon in the polymer with chain extension structure is more obvious to the electrolyte, which will cause a volume expansion in the binder, and increase the distance between the electrode materials; thus, the interface impedance is affected. Figure 7 shows the charge and discharge test for 100 cycles at 0.5 C. The PPC-based batteries have a more stable discharge capacity. The capacity stability of PPC-based batteries is better than that of PVDF batteries. This is due to the lower electrochemical impedance of PPC-based polymer binders, which results in low polarization and a high stable discharge capacity during cycling. The discharge capacity of the batteries using PPC/PPC-P as a binder are higher than E-PPC/E-PPC-P-based batteries. This is because their mobility decreases at high temperatures, which results in them having a smaller contact area with the LFP than PPC/PPC-P.

**Figure 8:**
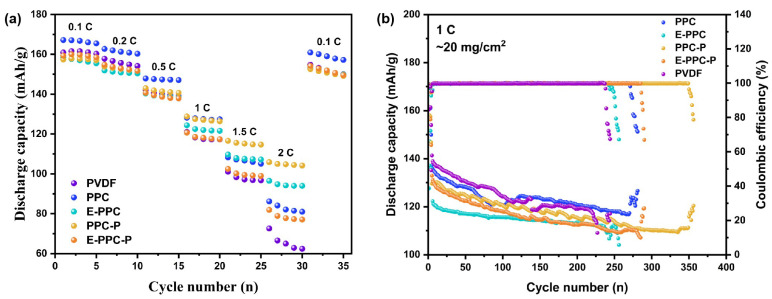
(**a**) Rate performance of different electrodes at various current densities (between 0.1, 0.2, 0.5, 1.0, 1.5, and 2.0 C); (**b**) The long-term cycle performance of different electrodes at 1 C.

Figure 8a shows the rate capacity performance of the batteries prepared using different binders. The discharge capacity of the batteries will decrease accordingly with the increase in the rate, thus showing a “step like” result. When the multiplier is reduced from 2 C to 0.1 C, the discharge capacity of the battery can quickly recover to the original value (nearly). The result shows that the PPC battery has a better rate capacity performance at low rates (0.1–1 C), while PPC-P battery has the optimal rate capacity performance at high rates (1–2 C), indicating that PPC-P has the strongest ability to inhibit ohmic polarization, which helps the battery to charge and discharge at a high rate. Figure 8b shows the long cycle properties of batteries at 1 C with different binders. It shows that PPC-based batteries have a more stable specific discharge capacity, a longer cycle life and a higher capacity for retention than PVDF batteries. Among them, E-PPC/E-PPC-P batteries show the most stable discharge capacity decay speed because they have better ionic conductivity, which ensures the stability of the electrode’s interfacial impedance. However, due to swelling, the capacity decay rate of batteries using chain-extension-structure binders is faster. The long cycle lives of all the PPC-based batteries are longer than that of PVDF battery. This is because the PPC-based polymers have a high tensile strength and satisfactory bonding effect, and a higher ionic conductivity coming from the rich polar carbonate groups. Among all the batteries, the PPC-P battery has the longest cycle life (350 cycles, 89% of capacity retention). The noises in the final stage of the long cycle numbers are because each battery is overcharged. This is because the load of the dry electrode (20 mg/cm^2^) is much higher than that of the conventional wet electrode (2 mg/cm^2^), which means that a large amount of Li^+^ becomes embedded and removed in the process of charge and discharge. This will lead to the creation of an uneven solid electrolyte interphase (SEI) film on the lithium anode. Adding more molding agents, such as LiNO_3_ and Fluoroethylene carbonate (FEC), to the electrolyte can effectively improve the cycling performance of the battery.

**Figure 9:**
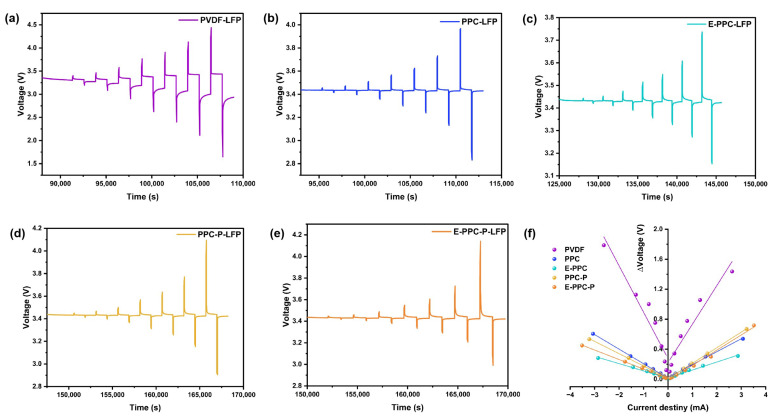
Potential responses of (**a**) PVDF-LFP, (**b**) PPC-LFP, (**c**) E-PPC-LFP, (**d**) PPC-P-LFP, (**e**) E-PPC-P-LFP batteries during DC-IR experiments at 30 °C; (**f**) plots of the change in voltage vs. current of different batteries.

**Figure 10:**
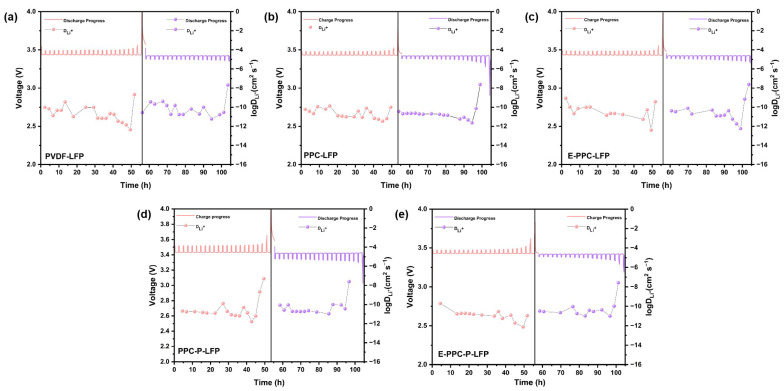
Voltage/D_Li_^+^–time curves of (**a**) PVDF-LFP, (**b**) PPC-LFP, (**c**) E-PPC-LFP, (**d**) PPC-P-LFP, (**e**) E-PPC-P-LFP batteries in GITT test.

Figure 9 shows the results of the DC-IR test for different batteries. The internal resistance of the batteries during charging and discharging processes were determined by linear regression analysis of the voltage and current changes diagram (Δ*V* − *I*). It shows that the slopes of the Δ*V* − *I* curves of PPC-based batteries are relatively low. This means that PPC-based binders with high a lithium-ion conductivity are superior to PVDF in reducing internal resistance. PPC-based batteries have a lower and more stable internal resistance than PVDF batteries. In addition, we used the GITT test to evaluate the stability of the ion diffusion coefficient of lithium ions in different electrodes (Figure 10). By calculating the Li-ion diffusion coefficient (D_Li_^+^) of each charging and discharging step, curves of the change of D_Li_^+^ could be drawn (see the curve connected by dots below each picture in Figure 10). Because the set voltage is reached in advance of the last charge or discharge, the charge and discharge are not complete, so there is a certain deviation in the calculation of the last D_Li_^+^ which can be ignored. It was found that the PPC-based electrodes have a more stable D_Li_^+^.

**Figure 11:**
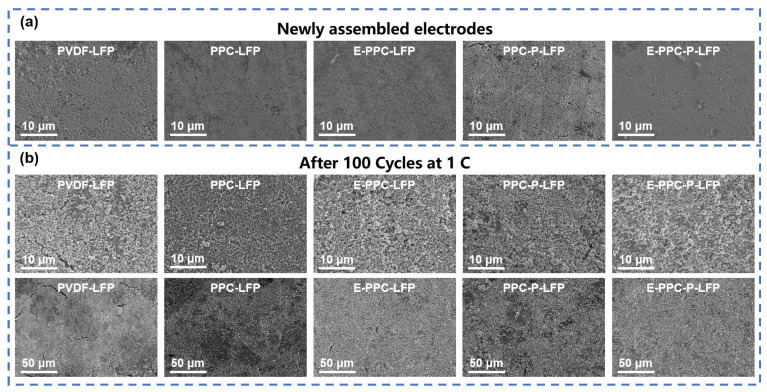
SEM images of different electrodes (**a**) Newly assembled electrodes before cycle and (**b**) after 100 cycles.

Figure 11 shows the surface morphology of different cathodes before and after 100 cycles using SEM. It shows that, compared with the PVDF electrode, the PPC-based electrodes are more compact before the circulation. After 100 cycles, the surfaces of the PPC-based electrodes are more complete and more compact. This is because the PPC-based binders are better dispersed in the dry electrodes, they can better maintain the integrity of the electrodes in the processes of charging and discharging.

## 4. Conclusions

In this work, we employed various PPC-based polymers as binders combined with the solvent-free method to prepare a dry LFP electrode. The PPC-based polymers are types of polymers synthesized using industrial waste gas CO_2_ as a raw material, with low production costs and no pollution to the environment. Using the solvent-free method, it is easy to prepare a high-areal-mass-loading electrode, but it also puts forward higher requirements for the dispersion ability and adhesive ability of the binders. The presence of a high proportion of heteroatoms in PPC-based polymers endows them with a higher adhesion force than PVDF in the dry electrodes. Meanwhile, the PPC-based binders can have good flow capacity at 150–200 °C. Thus, PPC-based polymers are well suited for use in dry electrode binders. In addition, due to the abundant polar carbonate groups in the PPC-based binders, they have higher Li^+^ conductivity than PVDF, and the benzene rings in PPC-P/E-PPC-P make their ionic conductivity even higher than PPC/E-PPC. The LFP|Li batteries were assembled using PPC-based binders with an areal mass loading of ~20 mg/cm^2^. The E-PPC/E-PPC-P batteries showed the most stable cycle performance. The PPC-P battery showed the longest cycle performance, with a retention rate of 89% after 350 cycles at 1 C. Moreover, the PPC-based binders have advantages in terms of the recycling and utilization of battery electrodes. Compared with PVDF, the decomposition temperature of PPC-based polymers is lower, and the decomposition products are harmless to the environment, which can be completely decomposed into water and carbon dioxide at 250–400 °C. The above performance is sufficient to prove the potential of PPC-based binders as commercial LFP electrode binders, or even as other commercial electrodes. This paper provides a class of low-cost and high-performance binders combined with the solvent-free method. It provides a new reference for the development of a commercial LFP electrode and dry electrodes.

## Data Availability

The original contributions presented in the study are included in the article, further inquiries can be directed to the corresponding authors.
